# Protein embedding based alignment

**DOI:** 10.1186/s12859-024-05699-5

**Published:** 2024-02-28

**Authors:** Benjamin Giovanni Iovino, Yuzhen Ye

**Affiliations:** Luddy School of Informatics, Computing and Engineering, Indiana University, 700 N. Woodlawn Avenue, Bloomington, IN 47408 USA

**Keywords:** Protein embedding, Protein sequence alignment, Smith-Waterman algorithm, Twilight zone

## Abstract

**Purpose:**

Despite the many progresses with alignment algorithms, aligning divergent protein sequences with less than 20–35% pairwise identity (so called "twilight zone") remains a difficult problem. Many alignment algorithms have been using substitution matrices since their creation in the 1970’s to generate alignments, however, these matrices do not work well to score alignments within the twilight zone. We developed Protein Embedding based Alignments, or PEbA, to better align sequences with low pairwise identity. Similar to the traditional Smith-Waterman algorithm, PEbA uses a dynamic programming algorithm but the matching score of amino acids is based on the similarity of their embeddings from a protein language model.

**Methods:**

We tested PEbA on over twelve thousand benchmark pairwise alignments from BAliBASE, each one extracted from one of their multiple sequence alignments. Five different BAliBASE references were used, each with different sequence identities, motifs, and lengths, allowing PEbA to showcase how well it aligns under different circumstances.

**Results:**

PEbA greatly outperformed BLOSUM substitution matrix-based pairwise alignments, achieving different levels of improvements of the alignment quality for pairs of sequences with different levels of similarity (over four times as well for pairs of sequences with <10% identity). We also compared PEbA with embeddings generated by different protein language models (ProtT5 and ESM-2) and found that ProtT5-XL-U50 produced the most useful embeddings for aligning protein sequences. PEbA also outperformed DEDAL and vcMSA, two recently developed protein language model embedding-based alignment methods.

**Conclusion:**

Our results suggested that general purpose protein language models provide useful contextual information for generating more accurate protein alignments than typically used methods.

**Supplementary Information:**

The online version contains supplementary material available at 10.1186/s12859-024-05699-5.

## Background

Sequence alignment is one of the most common bioinformatics tasks and has many downstream applications. Alignment is performed to reveal similar regions between sequences that may indicate that they have originated from the same ancestral sequence and have changed throughout evolutionary time. Despite mutations occurring, it is possible that homologous sequences still share a similar purpose. In the case of proteins, this means they may have similar structure, and subsequently similar function. However, protein sequences do not need to be homologous to look or perform the same, nor do they need the same amino acid content. Sequences with less than 10–12% pairwise identity, or aligned residue pairs, have been found to be homologous and have similar structures [16]. The process of aligning protein sequences has been more or less the same for over three decades since the creation of dynamic programming algorithms, like Needleman–Wunsch (NW) [14] and Smith-Waterman (SW) [1], to generate alignments with the most matching residue pairs, indicated by a high “alignment score”. These scores are calculated by penalizing gaps, or regions of sequences that do not match, and with scores given by substitution matrices, like BLOSUM [5], which indicate the likelihood of one residue being substituted for another based on observed mutations in homologous proteins. This method is fast and very accurate when two sequences have high pairwise identity. Accuracy is quickly lost when under 20–35% pairwise identity, a region of sequence alignments referred to as the “twilight zone” [3], which leads to problems in many downstream tasks, such as structure prediction and remote homology detection.

Recent methods have proposed using protein language models to turn protein sequences into a vector or set of vectors, a process referred to as embedding [15], and perform tasks that would normally be difficult with sequences that have low pairwise identity. Protein sequences, which are represented by a twenty character alphabet (twenty-four including a few rare amino acids), can be modeled as a natural language and use many of the tools developed for natural language processing [15]. Amino acids can be thought of as words in a sentence, performing particular roles in the overall function of a protein. Moreover, the same amino acid can perform different roles in different proteins, yet this nuance is not captured by its character identity. Deep language models, such as the original text-to-text transformer (T5) architecture [23], and modified architectures like BERT [2], are trained with a masked language modeling (MLM) objective. They are given unlabeled text as input, hide a certain percentage of the input, and then try to predict what the missing tokens are [2] based on the parts of the input that they can see. Being able to train these models on unlabeled data allows for them to be fed massive amounts of raw data and learn the underlying meaning and patterns of the language [15]. This approach can be applied to protein sequences where language models are trained on large protein databases, like UniRef50 [21], UniRef100 [21], and BFD [20] [19], and are able to learn about the “language of life” [4]. These language models can then be used to embed proteins, turning individual residues, or even entire proteins, into vectors with a number of features that represent their overall purpose and function in a protein, much more so than their simple character identity.

ProtTrans [4] is a collection of protein language models with various architectures that were trained with the goal of producing informative embeddings that could be applied to various downstream tasks. This goal differentiates ProtTrans from other protein language models, like AlphaFold [7] and ESMFold [10] which were trained with the end goal of predicting protein 3D structure from sequences. ProtT5-XL-U50 was the highest performing model of all the ProtTrans models with respect to their downstream tasks [4], and for this reason we decided to apply this particular model for our work. This model was trained with the original encoder-decoder T5 architecture, however only the encoder was used for their analysis because adding the decoder achieved no improvement for their experiments [4]. With three billion parameters, this model was first trained on BFD-100 and then fine-tuned on UniRef50 and saw over 7 billion proteins during training. Other groups have tried using embeddings from the ProtT5-XL-U50 model for their own goals with some successes, such as remote homology detection using nearest neighbor search on embedding spaces (knnProtT5) [17] and creating multiple sequence alignments (MSAs) as in the vcMSA algorithm [12]. In the knnProtT5 method, potential hits (many of which are false positives) found by k-nn search using average-pooled coarse-grained protein-level embeddings need to be aligned using Smith-Waterman and it was found that some of the homologs found by k-nn search were dropped because they could not be aligned adequately [17]. The findings in [17] clearly suggested a need for the development of an embedding-based local alignment method to use the full potential of embeddings based homology search and other applications.

In this paper, we developed an approach called PEbA (abbreviation of Protein Embedding based Alignment) for pairwise protein sequence alignment to fill in the gap. PEbA is based on a dynamic programming algorithm, just as the Smith-Waterman [1] local alignment algorithm. Instead of using a substitution matrix to score residue pairs, PEbA uses the similarity between the contextual embeddings of amino acids that are derived from a protein language model. We note that PEbA is different from vcMSA, which is based on clustering and ordering amino acid contextual embeddings to produce multiple sequence alignments [12], but we were still able to compare pairwise alignments generated by vcMSA and PEbA. We also compared PEbA to three other alignment methods: BLOSUM scored aligments, DEDAL, and FATCAT. DEDAL is a deep learning model made specifically to align protein sequences with the goal of producing more accurate alignments and alignment scores for remote homologs [11]. FATCAT [9, 24] is a structural alignment algorithm designed to compare protein structures and serves as a comparison for methods that use three-dimensional structural information, which, in principle, can serve as the upper bound of accuracy for sequence-based alignments. We also compared PEbA with embeddings produced by ProtT5-XL-U50 and ESM-2. We found that PEbA with ProtT5 embeddings created substantially more accurate alignments than BLOSUM, outperformed vcMSA, was able to more accurately align longer sequences than DEDAL, and was nearly identical to structure-based alignments.

## Methods

### Protein language models

The PEbA program defaults to using the ProtT5-XL-U50 model to embed the sequences it is given. Protein sequences are embedded by tokenizing each residue and using the weights from ProtT5’s last hidden layer to extract a vector of 1024 dimensions for each token. Padding and special tokens were removed so the number of vectors matched the number of residues for each sequence. PEbA can either embed sequences on the fly or take embeddings as an input, as long as the embeddings are 1D arrays with the same length as the sequence. We embedded every sequence prior to alignment in our testing. With the embeddings on hand, the only significant difference between PEbA and alignment with a substitution matrix is calculating the cosine similarity between each pair of vectors, as opposed to looking up substitution scores in a table. There is a second checkpoint of ProtT5-U50 with 11 billion parameters, ProtT5-XXL-U50, that we considered using, but in their analysis [4], this model did not perform better than ProtT5-XL-U50, which has three billion parameters. With no discernible difference between ProtT5-XL and ProtT5-XXL except for the latter requiring more time to produce embeddings, we performed our work using ProtT5-XL.

We also tested embeddings produced by one of the ESM-2 [10] checkpoints, specifically ESM2-T36-3B-UR50D. ESM-2 has several different checkpoints to choose from, including one with 15 billion parameters (the largest protein language model to date), but we chose the checkpoint with three billion parameters to match the number of parameters in ProtT5-XL-U50. These ESM-2 models were trained with a masked language modeling objective just like ProtT5, however they used an encoder-only architecture during training [10], whereas ProtT5 used an encoder-decoder architecture. ESM-2 models also saw significantly less protein sequences during training, around 65 million unique sequences, while ProtT5 saw over seven billion sequences. The differences in architecture and in the number of training examples may help explain why PEbA with ProtT5 embeddings outperformed PEbA with ESM-2 embeddings. ESM-2-T36-3B-UR50D outputs embeddings with 2560 dimensions, over twice that of ProtT5-XL-U50.

### Protein embedding based alignments

Given two protein sequences X and Y, their alignment can be computed using a dynamic programming algorithm, similar as the Smith-Waterman (SW) algorithm [1]). Instead of using substitution matrix such as BLOSUM to score the matching of two amino acids, our approach uses a scoring function that is defined according to the cosine similarity between the embedding of respective amino acids (e.g., $x_i$ in X and $y_j$ in Y) in the protein sequences, as shown in the following equation:

1 $$\begin{aligned} \delta (x_i, y_j) = f(cosine(Emb(x_i), Emb(y_j)) \end{aligned}$$

where $\delta (x_i, y_j)$ is the matching score between the two vectorized residues $x_i$ and $y_j$, $Emb(x_i)$ and $Emb(y_j)$ are the contextual embeddings of residue $x_i$ and $y_j$ derived from a protein language model, respectively.

We empirically tried different functions to compute the matching score based on the cosine similarity of embeddings. We found that a function that simply multiplies the cosine similarity by 10, and together with the standard, position independent gap penalties (-11 and -1 for gap opening and gap extension, respectively) gave good performance, i.e.,

2 $$\begin{aligned} \delta (x_i, y_j) = 10 \times cosine(Emb(x_i), Emb(y_j)) \end{aligned}$$

### Benchmark alignments

We used the alignments collected in BALiBASE3 to test PEbA. BAliBASE 3 contains 10 different references, each one containing a particular group of protein sequences. Each reference has a dozen or more manually curated Multiple Sequence Alignments (MSAs) made from three dimensional structure comparison [22]. The references of interest in this project include references (abbreviated as RV) 11 and 12, which contain “equi-distant sequences with 2 different levels of conservation” [22], and RV911, RV912, and RV913, which contain sequences with linear motifs. Importantly, RV11 and RV911 contain sequences with less than 20% sequence identity, well within the twilight zone of sequence alignment. RV12 and RV912 contain sequences with 20-40% sequence identity, and RV913 with 40–80% sequence identity. This collection of references allowed us to test PEbA on sequences that are typically difficult to align, as well as sequences that substitution matrices should align accurately. We extracted all pairwise alignments and the corresponding sequences from the MSAs for our testings for a total of over 12,000 alignments. More information about each of these references can be found in Table 1.

**Table 1:** Information on each BAliBASE reference used

	RV11	RV12	RV911	RV912	RV913
# of MSA’s	38	44	29	27	27
# of PWA’s	943	2335	5816	1038	2312
Avg # of Seq	7	9	15	8	10
Avg Seq Length	309	387	702	462	501

# of MSA’s: the number of multiple sequence alignments in each reference. # of PWA’s: the total number of pairwise alignments that were extracted from each MSA. Avg Seq: the average number of sequences in each MSA. Avg Seq Length: the average sequence length for all sequences found in each reference

### Compared methods

Before comparing PEbA to other alignment methods we tested various parameters. The SW local alignment algorithm was compared to the NW global alignment algorithm using our cosine similarity scoring function and it was found that the SW alignment algorithm performed better on average for every reference (see Additional file 1: Table S1 and Fig. S1 for the comparison). We also compared PEbA with ProtT5 embeddings to PEbA with ESM-2 embeddings and found that PEbA performed best with ProtT5 embeddings (more information found in the Results section). Other parameters, such as gap penalties and the distribution of cosine similarity scores, were also tested. The best performing combinations were left as default settings in the program.

We first compared PEbA with substitution matrix based alignments. We compared local alignments generated with BLOSUM and PFASUM [8] to determine which matrix produced more accurate alignments for sequences with low pairwise identity. BLOSUM62 (as implemented by the ‘blosum’ package for python) produced better alignments compared to PFASUM60 and BLOSUM45 scored alignments, so we used BLOSUM62 when comparing PEbA to substitution matrix-scored alignments.

We then compared PEbA to DEDAL, or Deep Embedding and Differentiable Alignment, a model that specifically generates pairwise alignments. DEDAL is both an encoder-only transformer and parameterizer trained on 30 million unique protein sequences from UniRef50 with a masked language modeling objective [11]. It was also trained on pairs of homologous sequences with known alignments from the Pfam-A seed database [13]. Once trained, DEDAL encodes a pair of sequences with the transformer and computes gap and substitution scoring matrices with the parameterizer that are specific for a pair of sequences. It then finds the optimal local alignment using the Smith-Waterman algorithm. We downloaded DEDAL from their public github reposity at https://github.com/google-research/google-research/tree/master/dedal. All its results reported in this paper were derived using default settings of the program.

We also compared PEbA to vcMSA, or Vector-clustering Multiple Sequence Alignment, a novel multiple sequence alignment algorithm that aligns proteins based on the clustering and ordering of protein language model embeddings [12]. This algorithm contains eight different steps, including the generation of protein embeddings, and was reported to perform better than previously developed methods (including T-Coffee and MAFFT-GINSI), particularly for low pairwise identity alignments. We installed vcMSA from their public github repository at https://github.com/clairemcwhite/vcmsa. No default parameters were altered to get the results reported in this paper.

Lastly, we compared PEbA to FATCAT [9, 24], a structural alignment algorithm. FATCAT differs from the other methods used in this paper in that it requires three-dimensional structural information taken from PDB files. Given that protein structures are more conserved than their sequences [6], it would make sense that structural alignment tools create more accurate alignments than sequence (or sequence derived, such as an embedding) alignment tools, and thus make a useful comparison.

### Assessment of alignment quality

To assess the quality of the alignments derived by different programs, we compared derived alignments against reference alignments benchmarked in BAliBASE. We used Sum-of-Pairs (SP) score and F1 score to quantify the quality of the alignments as compared to the reference. These metrics were used in previous studies to assess the alignment quality [11, 18]. Specifically, SP is the proportion of aligned residue pairs that are found in both the reference alignment and the test alignment (excluding pairs with gaps). In the case of pairwise alignments, SP score and Total Column (TC) score are identical because each column in the alignment contains only one pair, but we will refer to it as SP score. F1 score is the harmonic mean of the recall and precision of the alignment, where the recall is the percentage of alignment columns in the reference alignment (the ground truth alignment) that are also found in the test alignment, and the precision is the percentage of alignment columns found by a program that are in the ground truth alignment.

## Results

### Embedding based scoring function

**Fig. 1:**
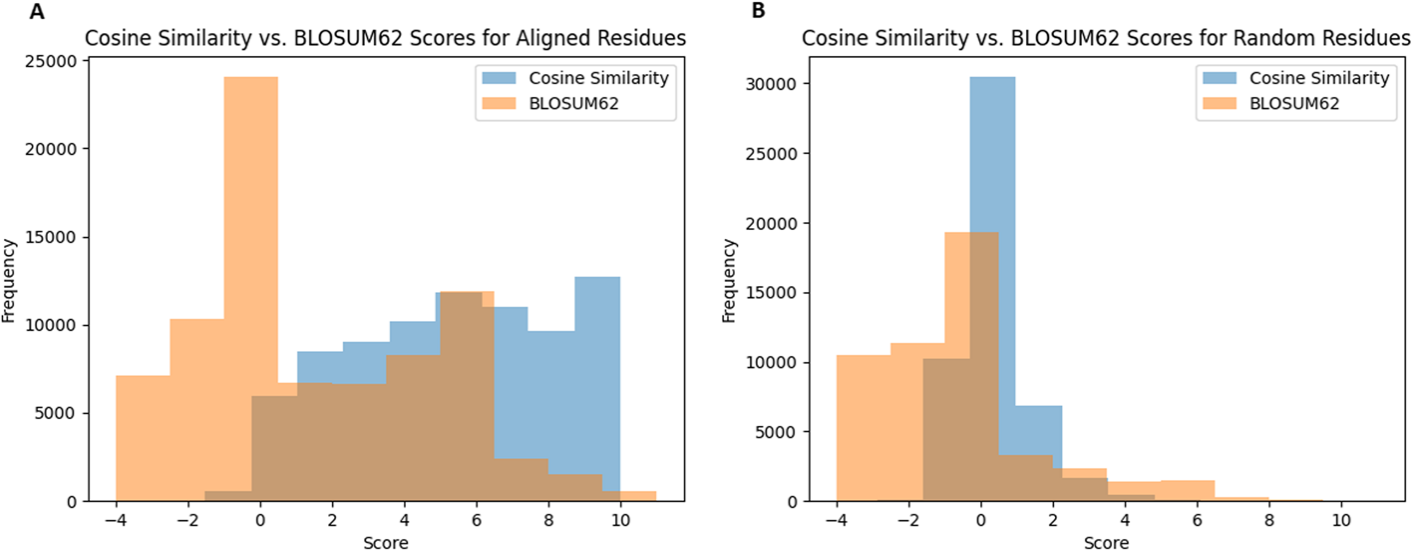
**A** Distribution of cosine similarity scores multiplied by 10 and BLOSUM62 substitution scores from aligned residue pairs in each BAliBASE reference. Each MSA was split into pairwise alignments. A random sample of 100 pairs from the first 5 pairwise alignments from each MSA were extracted so that one MSA did not have too significant of an effect on either distribution, as some MSA’s have much longer sequences or have alignments with much lower pairwise sequence identities than other MSA’s. **B** Distribution of cosine similarity scores multiplied by 10 and BLOSUM62 substitution scores from random residues in each BAliBASE reference. Four random residues were selected from five random sequences from each MSA and scored against each other. This number of samples was chosen to roughly match the number of samples in (**A**)

We derived a simple function (equation 2) to calculate the matching score between amino acids based on their contextual embeddings. It was inspired by examining the distributions of cosine similarities between aligned residues and random pairs of amino acids. Figure 1A shows the distribution of embedding-based matching scores to the distribution of BLOSUM62 substitution scores for residues that are aligned together in each reference. These distributions indicate that the scaled cosine similarity scores are on average more positive than the substitution scores based on BLOSUM62 substitution matrix, with the latter centered around zero, reflecting the fact that the benchmarks contain pairs of very low-similarity protein sequences. By contrast, Fig. 1B shows the same distributions but for randomly selected residues. As expected, both distributions center around 0, as random residue pairs are unlikely to be similar. When comparing the distributions between Fig. 1A and Fig. 1B, it is noteworthy that the BLOSUM62 distribution centers around zero (even for aligned pairs of residues), while the distribution of cosine similarity scores changes drastically from being centered around zero to being a uniform distribution. This contrast suggests that embedding-based scoring function can better capture the similarity of residues that have similar context despite being different amino acids.

We also tried a few other functions to calculate matching score based on embedding, but didn’t observe improvement of alignment quality. We therefore chose to use the simple function as shown in equation 2, and all the results below are based on this setting.

### PEbA performance

**Fig. 2:**
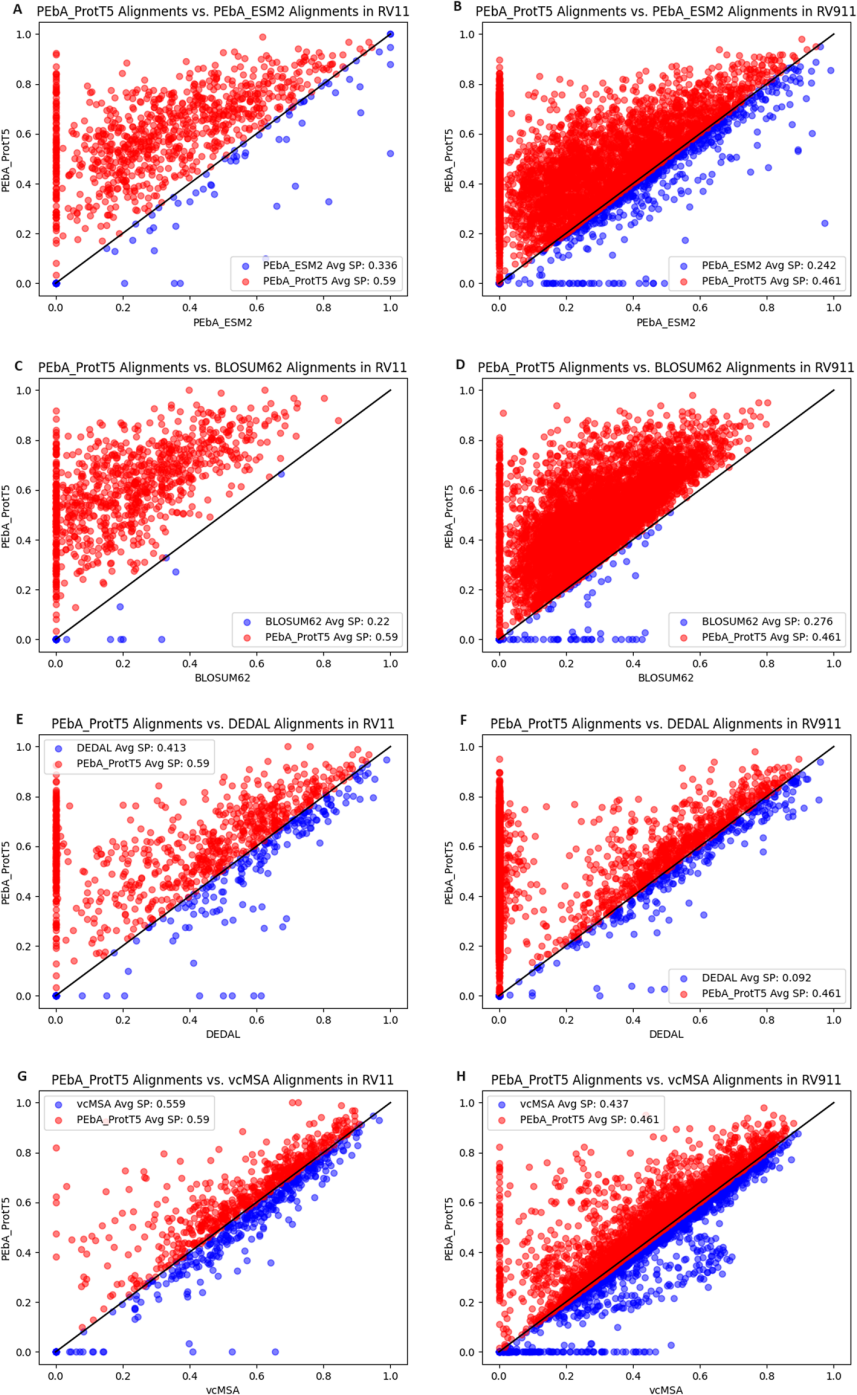
Comparison of the performance of PEbA and other tested methods on the reference alignments with low pairwise identity (<20%). Red points indicate an alignment where PEbA had a higher SP score relative to the reference than the other method (i.e., PEbA outperformed), and vice versa for blue points (i.e., PEbA underperformed). **A–B** PEbA with ProtT5 embeddings (PEbA_ProtT5) compared to PEbA with ESM-2 embeddings (PEbA_ESM2) for pairwise alignments from RV11 and RV911, respectively. **C–D** PEbA with ProtT5 embeddings compared to BLOSUM. **E–F** PEbA with ProtT5 embeddings compared to DEDAL. **G–H** PEbA with ProtT5 embeddings compared to vcMSA

PEbA with ProtT5 embeddings produced more accurate alignments (measured by SP score and F1 score) than every other tested method except for FATCAT. When referring to PEbA, it refers to SW alignments made using ProtT5 embeddings, unless otherwise specified. Table 2 summarizes the average SP scores for each method on each reference. Additional file 1: Table S2 shows the results in average F1 values, which revealed similar trends, though slight differences were observed. Figure 2 provides visualizations of the comparison.

**Table 2:** Comparison of alignment quality by the different methods on different sets of alignment benchmarks measured using the average SP score

	PEbA_ProtT5	PEbA_ESM2	BLOSUM62	DEDAL	vcMSA
RV11	0.590	0.336	0.220	0.413	0.559
RV12	0.844	0.715	0.626	0.648	0.828
RV911	0.461	0.242	0.276	0.092	0.437
RV912	0.755	0.633	0.594	0.377	0.685
RV913	0.940	0.900	0.874	0.203	0.922

FATCAT is not shown in this table because it was tested only on RV11 where it achieved an average SP score of 0.603

PEbA outperformed BLOSUM on average in every BAliBASE reference (RV) tested, particularly when pairwise identity between sequences was under 20% in RV11 and RV911 (see Figure 2A and B). Notably, even when pairwise identity increased, PEbA still outperformed BLOSUM, as seen by the average SP scores for RV913 in which pairwise identity between sequences ranged from 40-80%.

When using DEDAL in our tests, we observed that when the reference alignments were over 500 characters (residues and gaps), the comparison scores between DEDAL and the references decreased drastically. For references that contained longer alignments, especially RV911, the average SP score for DEDAL is even lower than the average SP score for BLOSUM, despite DEDAL producing much more accurate alignments than BLOSUM on shorter sequences. This may be because the alignment task of DEDAL was trained and tested on pairs of aligned domain sequences from Pfam-A [11]. Based on their supplementary methods, it appears that they had very few domains that were longer than 500 residues for this task. The lack of longer sequences in their training data may explain this drop in DEDAL SP scores.

PEbA outperformed DEDAL on average in every BAliBASE reference tested, but after noticing DEDAL’s shortcomings on longer alignments (e.g., > 500), we further compared PEbA and DEDAL using only the alignments less than 500 characters long. As shown in Table 3, PEbA and DEDAL performed comparably on sequences that share higher similarity (i.e., RV12, RV912 and RV913), but PEbA still outperformed DEDAL on alignments with lower identity (i.e., RV11 and RV911); PEbA achieved a SP score of 0.595 on RV11 whereas DEDAL achieved a SP score of 0.495. Figure 2C and D shows the SP scores from PEbA and DEDAL on RV11 and RV911, respectively. The red dots along the y-axis of these plots indicate all of the alignments where DEDAL failed to align any of the residue pairs from the reference. These alignments were likely outside of the range where DEDAL works well. We can see along the diagonal of the figure that DEDAL is still capable of producing accurate alignments, but it is the longer alignments that bring down the average SP score.

**Table 3:** Comparison of PEbA alignments and DEDAL alignments (in average SP scores) for alignments less than 500 characters in length

	PEbA	DEDAL
RV11	0.595	0.495
RV12	0.867	0.840
RV911	0.583	0.522
RV912	0.755	0.757
RV913	0.904	0.906

**Fig. 3:**
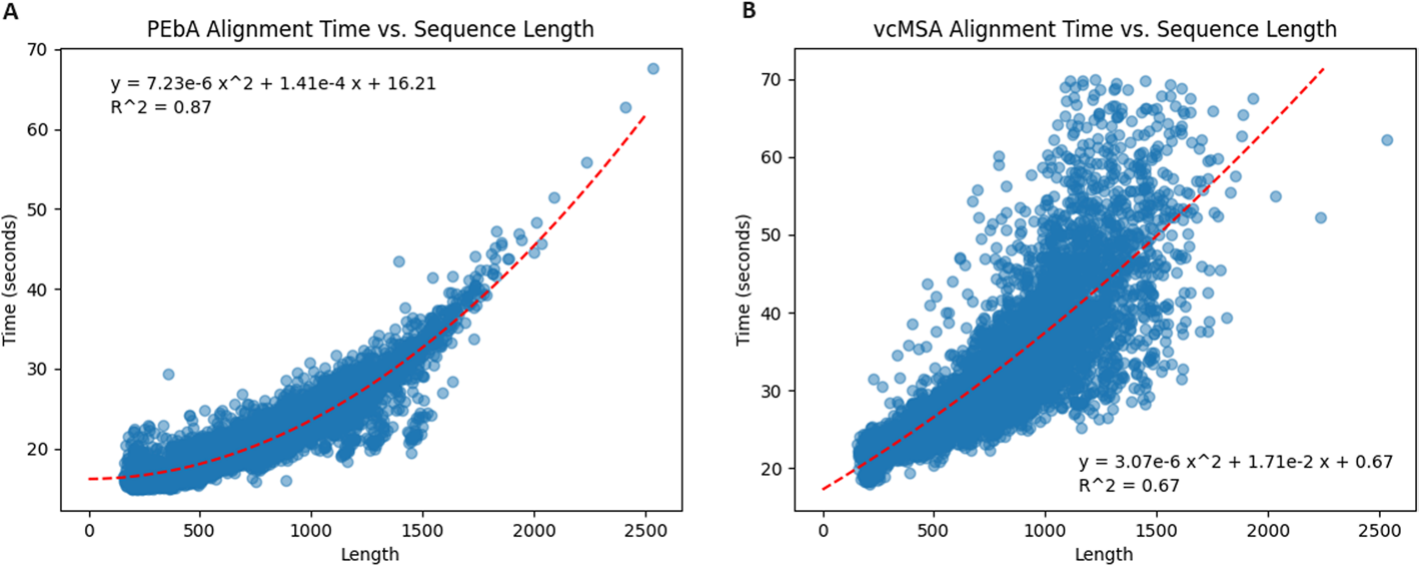
Comparison of the time it took for PEbA **A** and vcMSA **B** to align every pair from RV911. Each point is the average length of the two sequences in the alignment on the x-axis and the time it took to align them on the y-axis. The red dotted lines show the quadratic regression of the data points

vcMSA performed relatively well compared to every method we tested, but PEbA still performed better than vcMSA on average in every reference without any significant difference between alignment length or pairwise identity. We also note that PEbA is faster than vcMSA. For example, to align every pair in RV911 on the same computer (NVIDIA A40 GPU), it took PEbA about 22 seconds per alignment whereas it took vcMSA about 36 seconds per alignment. Most of this time involves loading the ProtT5 model, but vcMSA scales worse with alignment length than PEbA as seen in Fig. 3.

FATCAT was only tested on 905 of the 943 pairs in RV11 because this was the only reference to primarily use sequences with PDB identifiers. The average SP score for FATCAT alignments in RV11 was 0.603, marginally higher than PEbA’s average SP score of 0.597. Additional file 1: Figure S2 shows a graphical comparison similar to Fig. 2.

### Comparison of protein language models

PEbA with ProtT5 embeddings outperformed PEbA with ESM-2 embeddings on average in every BAliBASE reference tested, especially for sequences with low pairwise identity in RV11 and RV911, but the difference decreases as pairwise identity increases. PEbA with ESM2 embeddings (PEbA_ESM2) still produced more accurate alignments than BLOSUM in every reference except for RV911, however, vcMSA greatly outperformed PEbA_ESM2, indicating the effect that the protein language model has on the alignment power of PEbA. It is also worth mentioning that vcMSA averages the output from the final 16 layers of ProtT5 for each residue [12], yet PEbA better aligns two sequences by simply using the output from the final hidden layer.

### Effects of pairwise identity and sequence length on PEbA

**Fig. 4:**
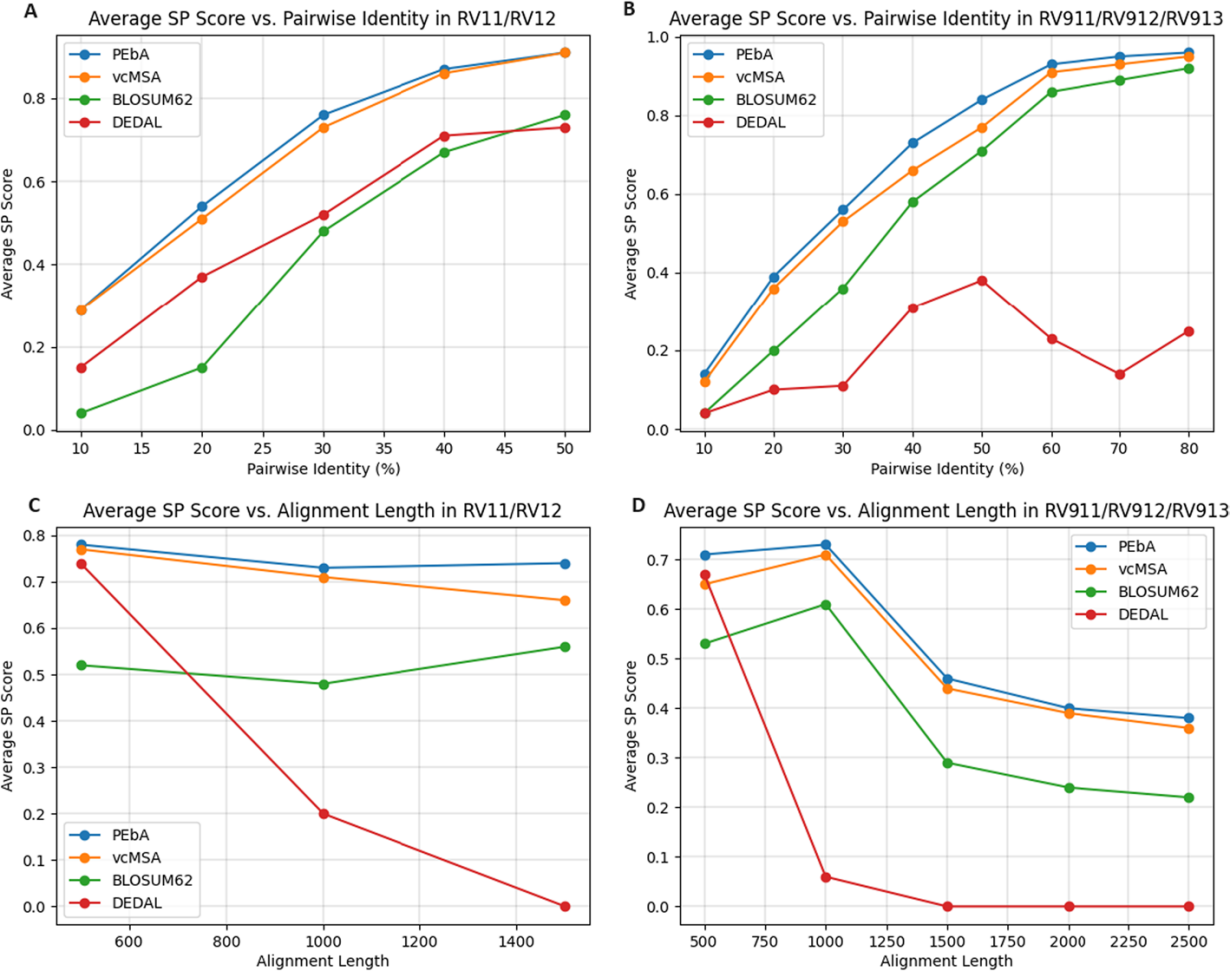
Average SP scores for PEbA, vcMSA, BLOSUM62, and DEDAL as percent identity or length increases for a given reference. Pairwise identity is binned as shown in Table 4, starting from 0-9% and increasing by 10% each bin. Length is binned as shown in Table 5, starting from 0-499 and increasing by 500 each bin. References 11/12 and 911/912/913 were grouped separately due to differences in number of sequences and average sequence length in each group that resulted in varying trends across the bins. **A** Average SP score as percent identity increases among sequences across RV11 and RV12. **B** Average SP score as percent identity increases among sequences across RV911, RV912, and RV913. **C** Average SP score as alignment length increases among sequences across RV11 and RV12. **D** Average SP score as alignment length increases among sequences across BAliBASE RV911, RV912, and RV913

We grouped together pairwise alignments from each reference by pairwise identity and length to see how these factors changed the performance of PEbA. Table 4 shows the average SP scores for PEbA and BLOSUM62, respectively, per similarity range. For sequences with 0-9% pairwise identity, PEbA performs 7 times better than BLOSUM62 in RV11 and 4 times better than BLOSUM62 in RV911. The magnitude of difference between PEbA and BLOSUM62 decreases as pairwise identity increases, but the average performance of PEbA generally increases and always outperforms BLOSUM62. Figure 4 shows the comparison of the performance of PEbA, vcMSA, BLOSUM62, DEDAL for each range of pairwise identity and alignment length. Since RV11 and RV12 differ from RV911, RV912, and RV913 in the number of sequences and their lengths, we showed the comparisons in two groups, with RV11 and RV12 in one group and the rest in the other group.

Table 5 shows the average SP scores for PEbA and BLOSUM, respectively, per length range. As length increases, both the performance of PEbA and BLOSUM62 decrease, but most importantly there is no significant degradation in the performance of PEbA (for RV11/12) or the degradation is more moderate (RV911/912/913) as the length of the reference alignments increases and PEbA outperforms BLOSUM62 in every category. Both Table 4 and Table 5 have hyphens indicating there were less than 10 alignments within that range, so no average was calculated to avoid skewed values.

### Limitations of PEbA

In RV911, 138 out of the 5816 pairwise alignments produced by PEbA had a SP score of zero. Of these 138, 31 of the corresponding BLOSUM alignments had a SP score greater than zero. These cases are seen by the blue dots along the x-axis of Fig. 2B. These particular comparisons show the limitation of PEbA that it cannot always produce a useful alignment for sequences with low pairwise identity compared to BLOSUM, even though in a vast majority of sequences it can. Most of these pairwise alignments come from the same two multiple sequence alignments, BOX214 and BOX076. Between these two, PEbA had 113 alignments with a SP score of zero, 21 of which BLOSUM produced an alignment with a SP score greater than zero. Some of these alignments were manually inspected and the PEbA alignments appeared to be stringent with it’s gap placement, whereas the BLOSUM alignments were much more lenient.

Of these 138 PEbA alignments, 115 of the corresponding vcMSA alignments had a non-zero SP score (seen by the blue dots along the x-axis of Fig. 2H), although the average SP score among these alignments is 0.13, much lower than either PEbA or vcMSA’s average SP score in RV911. Since vcMSA is a global alignment method and is likely to contain more residue pairs than a local alignment, it is possible that vcMSA, by nature of having more pairs to compare against the reference alignment, has more chances to land hits.

**Table 4:** Comparison of PEbA alignments and BLOSUM based alignments (in average SP scores) for each pairwise identity range

	0-9 (%)	10-19 (%)	20-29 (%)	30-39 (%)	40-49 (%)
RV11	0.29^a^/0.04^b^	0.55/0.15	0.71/0.39	–^c^	–
RV12	–	–	0.78/0.51	0.87/0.67	0.91/0.76
RV911	0.14/0.04	0.39/0.20	0.53/0.34	0.67/0.50	–
RV912	–	–	0.75/0.54	0.75/0.60	0.78/0.64
RV913	–	–	–	–	0.89/0.76

^a^: PEbA; ^b^: BLOSUM62; ^c^: data not shown when there are fewer than 10 pairwise alignments within that range of pairwise identity

**Table 5:** Average SP scores for PEbA/BLOSUM for each alignment length range

	0-499	500-999	1000-1499	1500-1999	2000-2499
RV11	0.59^a^/0.22^b^	0.57/0.22	–^c^	–	–
RV12	0.87/0.65	0.80/0.58	0.75/0.59	–	–
RV911	0.58/0.34	0.47/0.29	0.45/0.27	0.40/0.24	0.38/0.22
RV912	0.75/0.61	0.76/0.59	0.67/0.55	–	–
RV913	0.90/0.83	0.95/0.89	0.87/0.79	–	–

^a^: PEbA; ^b^: BLOSUM62; ^c^: data not shown when there are fewer than 10 pairwise alignments within that range of alignment length

### Case studies

Given that the BAliBASE benchmark alignments were curated based on 3D structure, PEbA’s better comparison scores to the benchmarks than other methods shows that PEbA aligns proteins based on structure more than character identity, presumably because of the structural information contained in the embeddings from the protein language models. Language models internalize the underlying patterns in protein sequences in order to predict amino acids during training, and with sequence determining structure, their embeddings must contain structural information. We used FATCAT [9] to showcase the 3D structural superpositions of protein pairs where PEbA aligned them nearly identical to the benchmark.

**Fig. 5:**
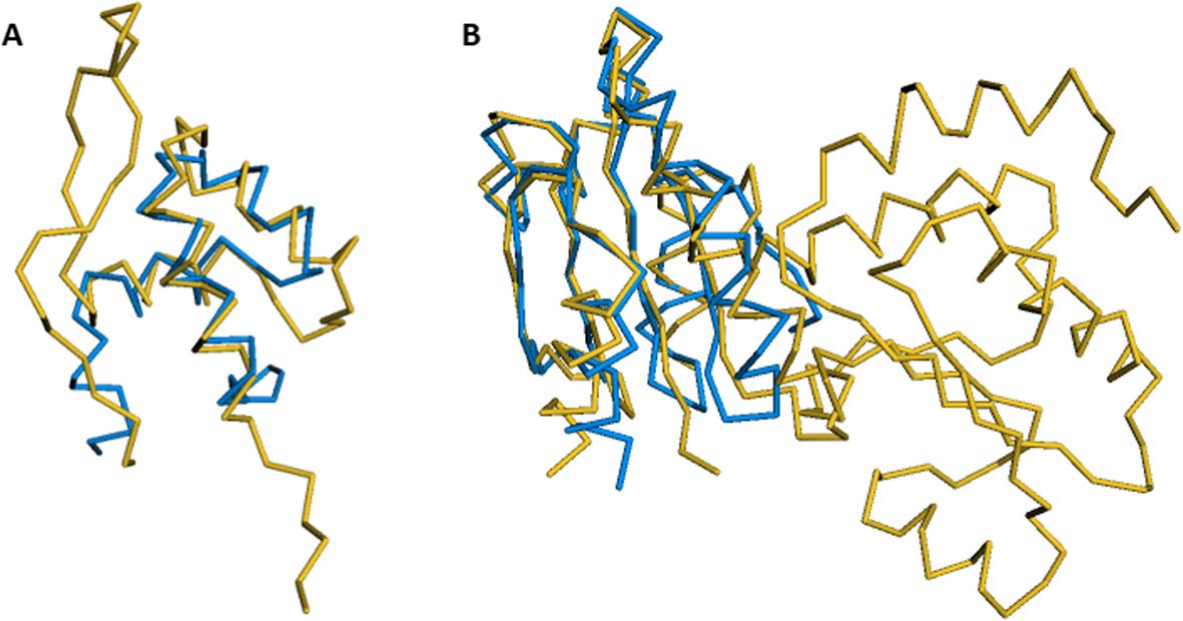
Examples of protein pairs that are aligned well by PEbA. **(A)** Proteins from RV11: 1tc3C (blue) and 1aoy (yellow) as shown in FATCAT superposition. **(B)** 1thx (blue) and 1a8lA (yellow)

Figure 5A shows the structural superposition of a transposase from *Caenorhabditis elegans* (PDB code: 1tc3, chain C) and the N-terminal domain of *Escherichia coli* arginine repressor (PDB code: 1aoy). These proteins show very similar structures, despite having only 11% pairwise identity. PEbA was able to generate an alignment with a SP score of 0.922 (i.e., more than 90% of the aligned positions by PEbA matched with the aligned pairs in the reference alignment), whereas BLOSUM generated an alignment with a SP score of 0 (the alignments don’t match at all). PEbA also outperformed DEDAL and vcMSA, which generated an alignment with a SP score of 0.431 and 0.373, respectively. DEDAL performed best with shorter sequences in each reference, but even in this example where 1tc3_C is 51 residues and 1aoy is 79 residues long, it could not produce an accurate alignment.

As another example, Fig. 5B shows the structural superposition of proteins 1thx (thioredoxin-2, with 108 residues) and 1a8lA (disulfide oxidoreductase, 226 residues). These sequences have 16% pairwise identity in the benchmark alignment. PEbA generated an alignment with a SP score of 0.860, whereas BLOSUM and DEDAL couldn’t align them at all both with a SP score of 0. vcMSA’s alignment for this pair had a SP score of 0.486.

## Discussion

Using ProtT5-XL-U50 to embed protein sequences and then using the cosine similarity between these embeddings to score each residue pair during local alignment proved to be a much more effective scoring method than using the substitution scores from BLOSUM. PEbA produces more accurate alignments than BLOSUM, on average, for every set of benchmark alignments. PEbA performs extremely well on alignments with less than 20% pairwise identity compared to BLOSUM and manages to maintain this increased performance as pairwise identity and alignment length increases.

Our method also proved to be more effective than two other protein language model embedding-based alignment methods, vcMSA and DEDAL, although vcMSA is much closer to PEbA in terms of average SP score for each reference than DEDAL. vcMSA’s performance is nearly identical to PEbA when using the NW global alignment algorithm (results for which can be found in the Additional file 1), but PEbA using the SW local alignment algorithm produces more accurate alignments in our testing. PEbA also has a clear time advantage when compared to vcMSA. We would expect our scoring method to be easily implemented into more optimized dynamic programming implementations, both for pairwise and multiple sequence alignments, which would increase the already existing difference in average time. However, the accuracy of MSA’s produced by PEbA’s scoring function remains to be seen.

The embeddings from ProtT5 clearly contain more information about each residue than its character identity, not just because of PEbA’s performance in comparison to BLOSUM, but also because PEbA nearly reaches the accuracy of FATCAT, which is encouraging considering that structural-based alignments tend to be considered more accurate than sequence-based alignments. Furthermore, since PEbA with ProtT5 embeddings and vcMSA (which also uses ProtT5) both greatly outperformed PEbA with ESM-2 embeddings and DEDAL, which produces its own embeddings, ProtT5 appears to generate the most informative embeddings of the three models for the task of alignment, further validating the observations of the ProtTrans [4] team. The success of PEbA with ProtT5 embeddings compared to ESM-2 embeddings can possibly be explained by the the sheer number of protein sequences that ProtT5-XL-U50 saw during training compared to ESM-2. The ProtTrans team noticed that more training, not necessarily more parameters, predicated more informative embeddings. It may also be explained by the way ProtT5-XL-U50 was trained; initially on a large and redundant database like BFD, and then fine tuned on a smaller and more refined database like UniRef50 [4]. ESM-2 trained their model solely on sequences from UniRef50 and saw over 60 million protein sequences during training [10], whereas ProtT5-XL-U50 saw over 7 billion. Future work could include using different checkpoints of ProtTrans models, or of other protein language models, to determine if model size impacts the performance of PEbA.

PEbA is, on average, able to produce more accurate alignments than BLOSUM even when sequences increase in length and sequences with high similarity. Depending on the need, BLOSUM may still be desirable for sequences with high pairwise similarity because PEbA needs embeddings as the input. However, if embeddings for two sequences already exist (precalculated), PEbA is not so much slower that it would be unreasonable to produce a pairwise alignment of interest if accuracy is more desirable than speed.

We noticed that the cosine similarity between the ProtT5 embeddings for the first few residues of each sequence was consistently much higher than the cosine similarity between embeddings of most other residues. This could be due to the language model focusing too much on the position of the initial residues as opposed to their identity and context within the sequence. We attempted to remedy this issue by using BLOSUM scoring for the first couple of residues in each sequence. This change to the scoring method results in either the same average alignment comparison score across each reference, or a slightly lower average score, so we kept using the cosine similarity between every single embedding during alignment.

Even though PEbA produces more accurate alignments on average than BLOSUM, there are still individual alignments where BLOSUM performs better. We tried tuning the distribution of cosine similarity scores, different gap scores, and using BLOSUM scoring for the first few residues in each sequence. There were some slight increases to PEbA’s average SP score score in certain references, but no such increase that was worth implementing permanently. The biggest increase in performance in this area will likely be embeddings from a larger model that is trained on both a higher number of and a more diverse set of protein sequences. With the exploding popularity of language models, newer and better protein language models will no doubt be trained. More informative embeddings that more accurately depict the role of amino acids within a protein sequence should only improve the performance of PEbA.

Finally, we note that in order to make PEbA practical for homolog search we will need further develop a filtering process or indexing scheme as searching against large dataset of sequences using the PEbA algorithm will be too slow. On the other hand, PEbA can be integrated with embedding based tools such as knnProtT5 [17] as the aligner to align the potential hits.

## Conclusion

Our study shows that aligning protein sequences with PEbA produces more accurate alignments than aligning them with the typical substitution matrix scoring, particularly those with low sequence identity. It also performs better than two other protein language model-based alignment methods. PEbA’s performance should only improve with larger protein language models that are trained on a higher number of sequences and make further progress towards aligning sequences within the twilight zone.

### Supplementary Information

**Additional file 1.** Supplementary information (Table S1 and Table S2, Figure S1 and Figure S2). 

## Data Availability

The data underlying this article and the codes are available github at https://github.com/mgtools/PEbA. The multiple sequence alignment benchmarks are available at https://www.lbgi.fr/balibase/.

## Abbreviations

PEbA: Protein embedding based alignment
BLOSUM: Blocks substitution matrix
DEDAL: Deep embedding and differentiable alignment
vcMSA: Vector-clustering Multiple Sequence Alignment
T5: Text-to-text transformer
ESM: Evolutionary scale modeling
MLM: Masked language modeling
BFD: Big fantastic database
NW: Needleman–Wunsch
SW: Smith-Waterman
MSA: Multiple sequence alignment
RV: Balibase reference
PFASUM: Pfam substitution matrix
TC: Total column
SP: Sum-of-Pairs
FATCAT: Flexible structure alignment
PDB: Protein data bank

## References

[1] Altschul Stephen F, Gish Warren, Miller Webb, Myers Eugene W, Lipman David J (1990). Basic local alignment search tool. J Mol Biol.

[2] Devlin J, Chang M-W, Lee K, Toutanova K. Bert: Pre-training of deep bidirectional transformers for language understanding. Proceedings of NAACL-HLT 2019, 4171–4186

[3] Doolittle RF (1986). ORFS A A primer on how to analyze derived amino acid sequences.

[4] Elnaggar Ahmed, Heinzinger Michael, Dallago Christian, Ghalia Rehawi Yu, Wang Llion Jones, Gibbs Tom, Feher Tamas, Angerer Christoph, Steinegger Martin, Bhowmik Debsindhu, Rost Burkhard (2022). ProtTrans: toward understanding the language of life through self-supervised learning. IEEE Trans Pattern Anal Mach Intell.

[5] Henikoff S, Henikoff JG (1992). Amino acid substitution matrices from protein blocks. Proc Natl Acad Sci.

[6] Illergård Kristoffer, Ardell David H, Elofsson Arne (2009). Structure is three to ten times more conserved than sequence a study of structural response in protein cores. Proteins Struct Funct Bioinf.

[7] Jumper John, Evans Richard, Pritzel Alexander, Green Tim, Figurnov Michael, Ronneberger Olaf, Tunyasuvunakool Kathryn, Bates Russ, Žídek Augustin, Potapenko Anna, Bridgland Alex, Meyer Clemens, Kohl Simon A. A, Ballard Andrew J, Cowie Andrew, Romera-Paredes Bernardino, Nikolov Stanislav, Jain Rishub, Adler Jonas, Back Trevor, Petersen Stig, Reiman David, Clancy Ellen, Zielinski Michal, Steinegger Martin, Pacholska Michalina, Berghammer Tamas, Bodenstein Sebastian, Silver David, Vinyals Oriol, Senior Andrew W, Kavukcuoglu Koray, Kohli Pushmeet, Hassabis Demis (2021). Highly accurate protein structure prediction with AlphaFold. Nature.

[8] Frank Keul, Martin Hess, Michael Goesele, Kay Hamacher (2017). PFASUM: a substitution matrix from pfam structural alignments. BMC Bioinformatics.

[9] Li Zhanwen, Jaroszewski Lukasz, Iyer Mallika, Sedova Mayya, Godzik Adam (2020). FATCAT 2.0: towards a better understanding of the structural diversity of proteins. Nucleic Acids Res.

[10] Lin Zeming, Akin Halil, Rao Roshan, Hie Brian, Zhu Zhongkai, Wenting Lu, Smetanin Nikita, Verkuil Robert, Kabeli Ori, Shmueli Yaniv, dos Santos Allan, Costa Maryam Fazel-Zarandi, Sercu Tom, Candido Salvatore, Rives Alexander (2023). Evolutionary-scale prediction of atomic-level protein structure with a language model. Science.

[11] Llinares-López Felipe, Berthet Quentin, Blondel Mathieu, Teboul Olivier, Vert Jean-Philippe (2022). Deep embedding and alignment of protein sequences. Nat Methods.

[12] McWhite CD, Armour-Garb I, Singh M (2023). Leveraging protein language models for accurate multiple sequence alignments. Genome Res.

[13] Mistry Jaina, Chuguransky Sara, Williams Lowri, Qureshi Matloob, Salazar Gustavo A, Sonnhammer Erik L L, Tosatto Silvio C E, Paladin Lisanna, Raj Shriya, Richardson Lorna J, Finn Robert D, Bateman Alex (2020). Pfam: the protein families database in 2021. Nucleic Acids Res.

[14] Needleman Saul B, Wunsch Christian D (1970). A general method applicable to the search for similarities in the amino acid sequence of two proteins. J Mol Biol.

[15] Ofer Dan, Brandes Nadav, Linial Michal (2021). The language of proteins: NLP, machine learning and protein sequences. Comput Struct Biotechnol J.

[16] Rost Burkhard (1999). Twilight zone of protein sequence alignments. Protein Eng Des Sel.

[17] Schütze K, Heinzinger M, Steinegger M, Rost B (2022). Nearest neighbor search on embeddings rapidly identifies distant protein relations. Front Bioinform..

[18] Sievers Fabian, Wilm Andreas, Dineen David, Gibson Toby J, Karplus Kevin, Li Weizhong, Lopez Rodrigo, McWilliam Hamish, Remmert Michael, Söding Johannes (2011). Fast, scalable generation of high-quality protein multiple sequence alignments using clustal omega. Mol Syst Biol.

[19] Steinegger Martin, Mirdita Milot, Söding Johannes (2019). Protein-level assembly increases protein sequence recovery from metagenomic samples manyfold. Nat Methods.

[20] Steinegger M, Söding J (2018). Clustering huge protein sequence sets in linear time. Nat Commun.

[21] Suzek Baris E, Wang Yuqi, Huang Hongzhan, McGarvey Peter B, Wu Cathy H (2014). UniRef clusters: a comprehensive and scalable alternative for improving sequence similarity searches. Bioinformatics.

[22] Thompson Julie D, Koehl Patrice, Ripp Raymond, Poch Olivier (2005). BAliBASE 3.0: latest developments of the multiple sequence alignment benchmark. Proteins: Struct Funct, Bioinf.

[23] Vaswani A, Shazeer N, Parmar N, Uszkoreit J, Jones L, Gomez A N, Kaiser L, Polosukhin I. Attention is all you need. Adv Neural Inform Process Syst 2017;30

[24] Yuzhen Ye, Adam Godzik (2003). Flexible structure alignment by chaining aligned fragment pairs allowing twists. Bioinformatics.

