# Supplementary material for: Protein embedding based alignment
Source: BMC Bioinformatics. 2024 Feb 28;25:85. doi: 10.1186/s12859-024-05699-5 (PMC10900708; doi:10.1186/s12859-024-05699-5)
Supplement: Supplementary file 1 — Additional file 1. Supplementary information (Table S1 and Table S2, Figure S1 and Figure S2). [file 12859_2024_5699_MOESM1_ESM.pdf]

Supporting Information:
Protein Embedding based Alignment

Benjamin Giovanni Iovino[1] and Yuzhen Ye[1,*]

[1]Luddy School of Informatics, Computing and Engineering, Indiana University, 700 N. Woodlawn Avenue, Bloomington, IN 47408, USA

Global vs. Local PEbA_ProtT5

We compared the performance between the Needleman-Wunch (NW) global alignment algorithm and the Smith-Waterman (SW) local alignment algorithm, both with our cosine similarity scoring function. PEbA with the SW local alignment algorithm (local PEbA) outperformed PEbA with the NW global alignment algorithm (global PEbA) on average in every reference. Table S1 summarizes the average SP scores for both methods on each reference.

Notably there appear to be many cases where global PEbA is able to produce an informative alignment where local PEbA is not, indicated by the blue dots on along the x-axis in Figure S1. In RV911, most of these cases are found in BOX076 and BOX214. Some of the sequences from BOX214 are relatively long, sometimes over 2000 residues, so it is possible that there are several high scoring segments in the alignment and a non-accurate one is chosen for traceback. Sequences in BOX076 are generally of average length for sequences in RV911, but there are many cases where global PEbA is only slightly better than local PEbA, perhaps due to luck when the global alignment returns more pairs than local alignment and randomly hits a few correct pairs.

Comparison using F1 Score

Table S2 summarizes the average F1 scores for each method on each reference. PEbA outperformed all methods in all benchmarks, except that vcMSA marginally outperformed PEbA in average F1 score for RV911.

Table S1: Comparison of PEbA with the local alignment (SW) algorithm and PEbA with the global alignment (NW) algorithm in average SP scores.

	local_PEbA	global_PEbA
RV11	0.590	0.566
RV12	0.844	0.829
RV911	0.461	0.445
RV912	0.755	0.750
RV913	0.940	0.938

Table S2: Comparison of alignment quality by the different methods on different sets of alignment benchmarks measured using the average F1 score.

	PEbA_ProtT5	PEbA_ESM2	BLOSUM62	DEDAL	vcMSA
RV11	0.583	0.325	0.242	0.443	0.575
RV12	0.842	0.708	0.672	0.663	0.836
RV911	0.455	0.227	0.295	0.100	0.460
RV912	0.749	0.623	0.634	0.395	0.715
RV913	0.938	0.896	0.899	0.205	0.929

FATCAT is not shown in this table because it was tested only on RV11 where it achieved an average F1 score of 0.607.

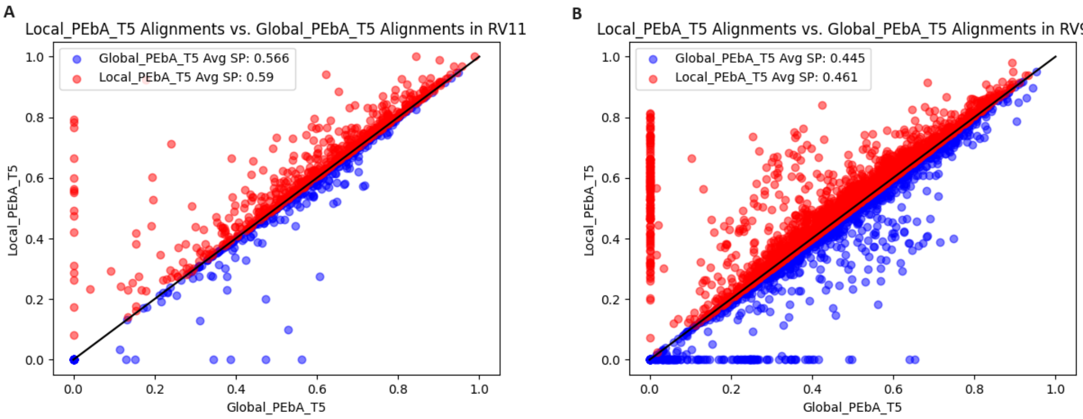

Figure S1: Comparison of the performance of PEbA with the local alignment (SW) algorithm **(A)** and PEbA with the global alignment (NW) algorithm **(B)** on the references with low pairwise identity (<20%). Red points indicate an alignment where local_PEbA had a higher SP score relative to the reference than global_PEbA, and vice versa for blue points.

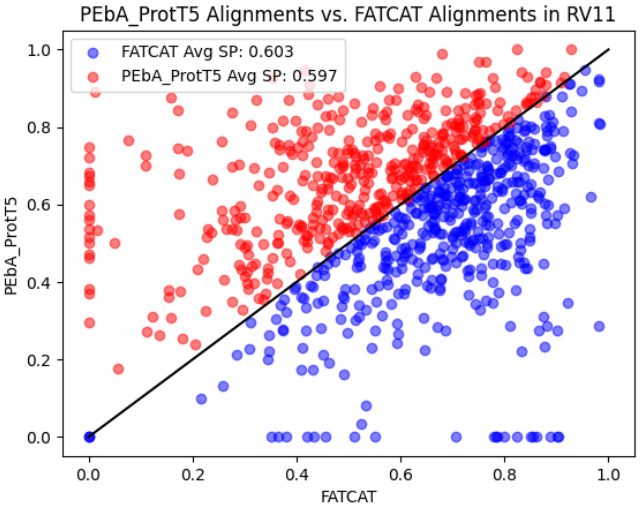

Figure S2: Comparison of the performance of PEbA and FATCAT on RV11.
